# Trends in seasonal influenza vaccine coverage of target groups in France, 2006/07 to 2015/16: Impact of recommendations and 2009 influenza A(H1N1) pandemic

**DOI:** 10.2807/1560-7917.ES.2018.23.48.1700801

**Published:** 2018-11-29

**Authors:** Pierre Verger, Lisa Fressard, Sébastien Cortaredona, Daniel Lévy-Bruhl, Pierre Loulergue, Florence Galtier, Aurélie Bocquier

**Affiliations:** 1 Aix-Marseille Université, IRD, AP-HM, SSA, VITROME, IHU-Méditerranée Infection, Marseille, France; 2 ORS PACA, Observatoire Régional de la Santé Provence-Alpes-Côte d'Azur, Marseille, France; 3 INSERM, F-CRIN, Innovative Clinical research Network in vaccinology (I-Reivac), GH Cochin Broca Hôtel Dieu, Paris, France; 4 Santé publique France, Direction des maladies infectieuses, Saint-Maurice, France; 5 Université Paris Descartes, Sorbonne Paris cité, Paris, France; 6 Inserm CIC 1417, Paris, France; 7 Assistance Publique Hôpitaux de Paris, CIC Cochin-Pasteur, Paris, France; 8 CIC 1411, CHU Montpellier, Hôpital Saint Eloi, Montpellier, France

**Keywords:** influenza vaccines, coverage, trends, recommendations, segmented regression

## Abstract

**Background and aims:**

Seasonal influenza vaccination (SIV) uptake (SIVU) rates in France are below target. We (i) describe trends in French SIVU over 10 consecutive seasons among different target groups and (ii) examine the effects of the 2009 influenza A(H1N1) pandemic and the publication of new SIV recommendations in 2011 and 2013.

**Methods:**

Our study was based on records of vaccines delivered in community pharmacies for a permanent, representative sample of 805,000 beneficiaries of the French National Health Insurance Fund. For the first objective, we analysed SIVU rate trends among ≥ 65 year olds as well as among  < 65 year olds with each of the following conditions: diabetes, respiratory, cardiovascular, neuromuscular, or chronic liver disease. For the second goal, we computed segmented log-binomial regression analyses.

**Results:**

After the 2009 pandemic, except for the target group with liver diseases, where the difference was not statistically significant, SIVU fell significantly in all groups during the 2010/11 season, remaining relatively stable until 2015/16 in groups not targeted by new recommendations. Crude SIVU rates in 2015/16 were 48% (43,950/91,794) for ≥ 65 year olds and between 16% (407/2,565) and 29% (873/3,056) for  < 65 year olds depending on their condition. SIVU increased modestly after new recommendations were published, but only in patients newly eligible for a free vaccine voucher.

**Conclusions:**

Our results suggest: (i) a prolonged confidence crisis in SIV, initially impelled by the 2009 pandemic vaccination campaign; (ii) that new recommendations are ineffective without additional measures. Interventional research in this field is a priority.

## Introduction

In most high-income countries, seasonal influenza vaccination (SIV) is recommended as a direct protection for elderly individuals (generally those aged ≥ 65 years) and for people aged ≥ 6 months at clinical risk [1,2]. Vaccinating the people likely to infect them (healthcare professionals especially) is also recommended (indirect protection of those at risk).

In 2009, the European Council recommended that national plans be developed and implemented to reach an SIV uptake (SIVU) rate of at least 75% in each of the at-risk groups by the 2014/15 season [3], an objective already included in the 2004 French Public Health Policy Act [4]. In Europe, most countries have established publicly funded programmes to enable the target groups to obtain this vaccine free of charge [5]. 

In France, most influenza vaccinations are performed by private practitioners, but all the insurance funds that make up the National Health Insurance Fund (NHIF) send a voucher to members of the target groups identified as such (based on long-term illness (LTI) status) and ensured by them. The voucher enables these individuals to obtain the vaccine free of charge at the pharmacy, without a doctor's prescription. They must then make an appointment with either a doctor or a nurse for the vaccine administration. People in the target groups not identified in the NHIF databases (i.e. persons in a risk group but who do not have LTI status) can obtain a voucher from their doctor but this makes their pathway to vaccination more complex as it requires first a doctor's appointment to get a voucher for free vaccine, then going to the pharmacy for delivery, and then a second appointment for the actual vaccine injection.

Despite programmes to provide vaccination access, few countries have reached the objective of 75% uptake of the at-risk groups; Northern Ireland being an exception [1]. On the contrary, a notable trend towards reduction of SIVU has been observed in several countries among different at-risk groups: in the Netherlands, between 2008/09 and 2013/14, for the at-risk groups overall [6]; in Spain between 2008/09 and 2011/12 among those younger than 60 years with a chronic disease [7]; in France, between 2009/10 and 2011/12, in those aged ≥ 65 years and in some of the younger at-risk groups, such as those with diabetes [8,9]. SIVU rates in Canada are also far below the national target of 80% and decreased between 2006/07 and 2013/14 among those aged ≥ 65 years [10]. The causes of these observations are probably multiple and different from one country to another. They include (but are not limited to) media controversies about the effectiveness of the vaccine [6], epidemics that are less marked than in the past [6], and reactions to the vaccination campaign for the expected pandemic in 2009, especially in France, where this campaign was strongly decried [9]. To understand SIVU trends, a first question is how these evolve over the years and for different target groups. 

A further question is to what extent the publication of new influenza vaccination recommendations might affect SIVU rates. In France, new recommendations were introduced after the 2009 influenza A(H1N1) pandemic – in March 2011 and then in April 2013 (see the methods section) – to expand the list of chronic underlying diseases considered to pose a risk for influenza. The NHIF had already targeted some of these and had been sending annual vouchers to patients with such diseases for several years. In this group we can study the effect of the publication of the recommendations, separately from the effect of the voucher. For others (such as for persons with chronic liver diseases), the NHIF did not begin sending free vouchers until the new recommendations were published.

Our study sought to: (i) describe trends in SIVU over 10 consecutive seasons in France among different target groups (objective 1); and (ii) examine how they were affected by the 2009 influenza A(H1N1) pandemic (objective 2a) and the publication of new SIV recommendations (objective 2b).

## Methods

### Study design and data source

The study consisted of repeated cross-sectional assessments of SIVU in a dynamic cohort: the Permanent Sample of Beneficiaries (Echantillon Généraliste des Bénéficiaires, EGB). It was set up in 2005, by a national random sampling of one of every 97 persons affiliated with one of the three major national health insurance funds in France (salaried workers, agricultural workers and farmers, and self-employed workers) [11]. The sample is permanent, representative and open: every 3 months, new entrants (e.g. registered births) are added to the database; inversely, personal data of those considered to have exited (e.g. died) are nevertheless preserved in the database to permit their characterisation in longitudinal studies. At the moment of data extraction (August 2017), the EGB included 804,089 beneficiaries.

Data are updated monthly from the NHIF administrative databases and include age, sex, area of residence, insurance fund, reimbursement claims for laboratory tests performed or drugs purchased in the community (classified by Anatomical Therapeutic Chemical (ATC) codes) and status for LTI [12]. On request of their doctors, LTI status is granted to beneficiaries with long-term and costly diseases and this status exempts them from co-payments for any medical care associated with the disease(s) in question, regardless of their income level [11]. Since 2006, data regarding the diagnoses associated with admissions to a French public or private hospital, excluding military and psychiatric hospitals, are also available for all individuals in the EGB.

For this study, we extracted data for salaried workers and their covered family members (i.e. the children and non-working spouses of salaried workers) only (ca 86% of the French population), because people benefiting from other insurance funds were not included in the EGB until 2011.

Access to the EGB is regulated by law and authorised by the National Committee for Informatics and Privacy (Commission Nationale de l’Informatique et des Libertés), and data are anonymous. We obtained authorisation to access the EGB database from the French NHIF.

### Study population

**Figure 1:**
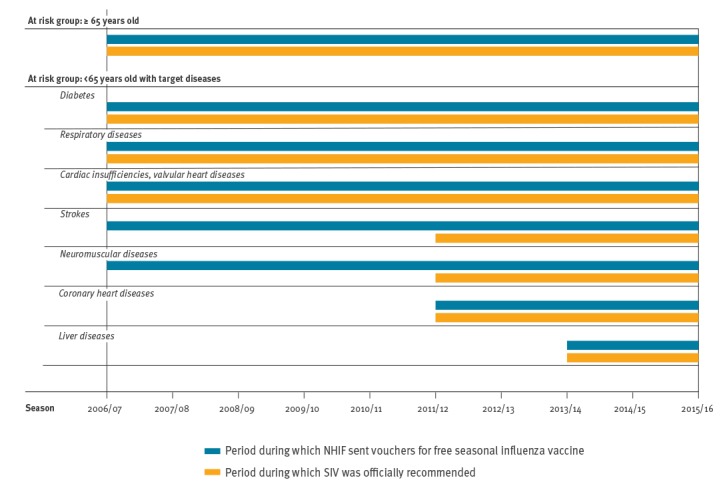
Availability of vouchers for free seasonal influenza vaccine (SIV) across influenza seasons and periods when SIV was recommended, according to at-risk group, France, 2006/07–2015/16

NHIF: National Health Insurance Fund; SIV: seasonal influenza vaccination.

The study population comprised most individuals eligible for SIV in at least one seasonal campaign between 2006/07 and 2015/16. Inclusion criteria were residence in mainland France at year *n* and coverage by the NHIF that same year; alive at the start of each campaign (1 September, *n*); age ≥ 65 years or with at least one of the five main chronic conditions specified in the 2015 French recommendations for SIV [13]: diabetes as well as respiratory, cardiovascular, neuromuscular (that is, involving neurological and/or muscular symptoms), and chronic liver diseases. Figure 1 reports the dates of publication of SIV recommendations and the periods when vouchers have been sent, according to disease subcategories. Overall, 95% of the individuals younger than 65 years who received a free vaccination voucher from the NHIF in 2015 have one of these five conditions. To identify targeted chronic conditions, we adapted algorithms published by the NHIF [14] based on LTI status, diagnoses at hospitalisation, and specific drugs or laboratory test reimbursement claims. This allowed us to identify the people targeted by the recommendations as accurately as possible (algorithms available on request). The NHIF principally uses LTIs to identify people who will be sent vouchers (Supplement 1).

### Statistical analyses

For both objectives, we stratified the analyses for two age categories (< 65 years; ≥ 65 years) because SIVU rates are much lower among those < 65 years old, and the factors associated with uptake may differ according to these categories [9].

#### Trends in French seasonal influenza vaccination uptake among different target groups 

For each season *n/n + 1*, we estimated SIVU rates as the ratio of individuals with a recorded SIV delivery (ATC codes J07BB02, J07BB03, J07CA, excluding A(H1N1) pandemic vaccines) between 1 September of year *n* and 31 March of year *n + 1* to the number of individuals in the corresponding group identified in the database in year *n*. Each SIV delivered in a community pharmacy is recorded in the NHIF database, including those delivered with a free voucher.

To describe the trends of SIVU rates, we standardised directly by age and sex, using the 2007 mainland France national census as the reference, to take into account population ageing and the varying age and sex distributions across target groups.

#### Impact of 2009 influenza A(H1N1) pandemic and new seasonal influenza vaccination recommendations on uptake trends 

To compare trends of SIVU rates before and after specific events – the 2009 influenza A(H1N1) pandemic (objective 2a), or publication of new recommendations (objective 2b) in March 2011 (for strokes, coronary heart diseases and neuromuscular diseases) and in April 2013 (liver diseases) – we computed segmented log-binomial Generalised Estimating Equation (GEE) regression models, using the binary variable ’SIVU (yes/no)’ as the outcome. 

A log-binomial approach allows us to model the probability of SIVU directly and to take within-subject correlations (repeated observations on individuals over more than one season) into account. Segmented regression analysis is an effective and robust method for assessing statistically the longitudinal effects of events on an outcome measure, instantaneously and/or in the long term [15,16]. Depending on the at-risk group, two to three ‘change points’ (i.e. specific points in time when an identifiable event may imply a change in trends) were considered: the World Health Organization’s (WHO) pandemic declaration [17], which led in France to the recommendation to launch a mass vaccination campaign (September 2009) [18]; the end of this campaign in 2010 after WHO announced the end of the pandemic [19], and the introduction of new SIV recommendations (in March 2011 and again in April 2013).

Among groups unaffected by the new SIV recommendations (people aged ≥ 65 years and those < 65 years with diabetes, respiratory diseases, or cardiac insufficiency/valvular heart diseases), we used the following model:

Log (*p*) = *β*_0_ + *Sex* + *Age_t_* + *β*_1_ × *T_t_* + *β*_2_ ×  *Pandemic* + *β*_3_ × *End_pandemic*  + *β*_4_ × *Time_after_pandemic_t,_*

where p is the probability of SIVU; ${Age}_{t}$ is age in years during season *t* (< 15, 15–34, 35–44, 45–54, 55–64, 65–74, 75–84, > 84); *T_t_* is a continuous discrete variable indicating the season number at time *t*, varying from 1 (2006/07 season) to 10 (2015/16 season); *Pandemic* is a dummy variable coded 0 before and 1 as of the start of the 2009 influenza A(H1N1) pandemic (first changepoint); *End_pandemic* is a similar dummy variable coding for the end of the pandemic (second change point); *Time_after_pandemic_t_* is a continuous variable counting the number of seasons at time *t* after the end of the pandemic, coded 0 before 2010/11 and increased by 1 at each subsequent new season.

Log-binomial models make it possible to estimate the relative risks of SIVU by exponentiating the regression coefficients. For this model, we estimated ${exp(\beta }_{1})$, the temporal trend of uptake rates over the pre-pandemic period (from 2006/07 to 2008/09); ${exp(\beta }_{1}+\beta_{2})$, the immediate change in rates after the start of pandemic in 2009 (that is, change in rates between seasons 2008/09 and 2009/10; first change point); ${exp(\beta }_{1}+\beta_{3}+\beta_{4)}$, the change in rates between seasons 2009/10 and 2010/11, after the end-of-pandemic statement in 2010 (second change point); and ${exp(\beta }_{1}+\beta_{4})$, the temporal trend after the end of the pandemic (from 2011/12 to 2015/16 seasons).

We applied similar models including additional change points for the groups targeted by new recommendations in 2011 or 2013. To assess the choice of GEE working correlation structure, we calculated the quasi-likelihood under the independence model criterion (QIC) and selected the model with the lowest QIC. The QIC statistic is available for GEE models and is similar to the Akaike information criterion (AIC).

All analyses were based on two-sided p-values, with p ≤ 0.05 indicating statistical significance, and were conducted with SAS, version 9.4.

## Results

Over the study period, 121,343 beneficiaries contributed to the analyses among the ≥ 65 year age group (including people who were < 65 years old in 2006 and became older during the follow-up); 103,542 people contributed to the analyses in the < 65 year old group. Characteristics of each group for the first and the last season of the study period are described in Table 1. We included between 22% and 76% additional persons (varying according to their chronic disease), compared with the numbers identified by tracking routine vouchers (Supplement 1).

**Table 1:** Distribution of the study population by sociodemographic characteristics and chronic diseases, for each target group, for the first and last season of the study period, Permanent Sample of Beneficiaries (EGB), France, 2006/07–2015/16

Characteristics	< 65 years old with target diseases^a^				≥ 65 years old			
	2006/07 (n = 37,802)		2015/16 (n = 39,147)		2006/07 (n = 72,595)		2015/16 (n = 91,794)	
	n	%	n	%	n	%	n	%
Women	17,045	45.1	17,953	45.9	43,132	59.4	52,989	57.7
**Age interval in years**								
0–14	7,855	20.8	5,756	14.7	NA	NA	NA	NA
15–34	5,552	14.7	4,996	12.8	NA	NA	NA	NA
35–44	5,116	13.5	4,698	12.0	NA	NA	NA	NA
45–54	8,051	21.3	8,976	22.9	NA	NA	NA	NA
55–64	11,228	29.7	14,721	37.6	NA	NA	NA	NA
65–74	NA	NA	NA	NA	36,294	50.0	47,269	51.5
75–84	NA	NA	NA	NA	26,496	36.5	29,272	31.9
≥ 85	NA	NA	NA	NA	9,805	13.5	15,253	16.6
**Targeted condition** ^b^								
None	NA	NA	NA	NA	45,536	62.7	53,788	58.6
Liver diseases	2,183	5.8	2,565	6.6	779	1.1	1,374	1.5
Neuromuscular diseases	1,823	4.8	2,225	5.7	759	1.1	1,533	1.7
Diabetes	9,428	25.0	13,389	34.2	10,437	14.4	17,622	19.2
Cardiac insufficiencies and valvular heart diseases	2,355	6.2	3,056	7.8	5,766	7.9	9,525	10.4
Strokes	1,373	3.6	1,877	4.8	2,719	3.8	4,154	4.5
Coronary heart diseases	4,116	10.9	5,102	13.0	10,007	13.8	12,926	14.1
Respiratory diseases	21,799	57.7	18,975	48.5	7,930	10.9	10,332	11.3
At least one other chronic condition	5,317	14.0	6,648	17.0	18,236	25.1	23,963	26.1

NA: not applicable.

^a ^Diabetes, respiratory, cardiovascular, neuromuscular and/or chronic liver diseases.

^b ^Each individual can suffer from several targeted conditions (e.g. a chronic respiratory disease and diabetes).

The total number of individuals considered in the Table was 175,038.

### Trends in seasonal influenza vaccination uptake rates (objective 1)

**Figure 2:**
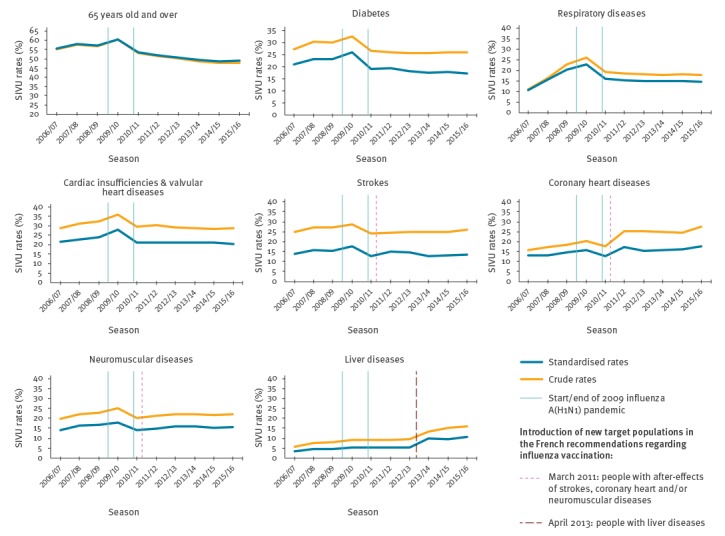
Trends in seasonal influenza vaccination uptake, crude and standardised rates, in eight at-risk groups, Permanent Sample of Beneficiaries (EGB), France, 2006/07–2015/16

Figure 2 depicts the trends in crude and standardised SIVU rates (corresponding values available in Supplement 2). The standardised and crude rates were similar for people aged ≥ 65 years (Figure 2). In contrast, the standardised rates in the < 65 year-old groups, were systematically lower than crude rates (7 percentage points (pp), on average); the shapes of the corresponding curves (crude and standardised) nonetheless remained similar in each group. Throughout the study period, crude SIVU rates were clearly and significantly (chi-squared test: p < 0.001) higher in the ≥ 65 year-old group (48% in 2015/16) than among the < 65 year-old groups with targeted conditions (from 16% to 29% in 2015/16, according to condition).

SIVU: seasonal influenza vaccination uptake.

The total number of individuals considered in the Figure was 209,467.

### Trends with respect to the 2009 influenza A(H1N1) pandemic 

In the seasons before the 2009 influenza A(H1N1) pandemic, segmented regressions (Table 2) showed a significant positive trend in standardised SIVU rates in all at-risk groups.

**Table 2:** Analysis of changes in the level and trends in seasonal influenza vaccination (SIV) standardised uptake rates after the 2009 influenza A(H1N1) pandemic and introduction of new SIV recommendations in March 2011 and April 2013, Permanent Sample of Beneficiaries (EGB), France, 2006/07–2015/16^a^

Events and trends	≥ 65 years old (n = 121,343)	< 65 years old with target diseases (n = 103,542)							
		Diabetes	Respiratory diseases	Cardiac insufficiencies and valvular heart diseases	Strokes^b^	Coronary heart diseases^b^	Neuromuscular diseases^b^	Liver diseases^c^	
	RR (95% CI)	RR (95% CI)	RR (95% CI)	RR (95% CI)	RR (95% CI)	RR (95% CI)	RR (95% CI)		RR (95% CI)
**Pre-2009 influenza A(H1N1) pandemic**									
Temporal trend^d^ over the pre-pandemic period (2006/07–2008/09)	**1.01 (1.01–1.02)**	**1.04 (1.03–1.06)**	**1.35 (1.32–1.37)**	**1.06 (1.02–1.09)**	**1.05 (1.00–1.10)**	**1.08 (1.04–1.12)**	**1.07 (1.02–1.12)**		**1.14 (1.04–1.24)**
**2009 influenza A(H1N1) pandemic**									
Immediate change after the start of pandemic (Jun 2009–2009/10)	**1.05 (1.04–1.06)**	**1.06 (1.02–1.10)**	**1.09 (1.05–1.14)**	**1.09 (1.02–1.18)**	1.03 (0.93–1.15)	**1.09 (1.00–1.18)**	1.07 (0.97–1.19)		1.09 (0.88–1.32)
Immediate change after the statement of end of pandemic (September 2010–2010/11)	**0.88 (0.87–0.89)**	**0.81 (0.78–0.84)**	**0.75 (0.72–0.79)**	**0.84 (0.78–0.90)**	**0.84 (0.76–0.93)**	**0.87 (0.81–0.94)**	**0.80 (0.73–0.88)**		0.98 (0.79–1.22)
Temporal trend^d^ after end of pandemic (2011/12–2015/16^e^)	**0.98 (0.97–0.99)**	1.00 (0.98–1.02)	0.98 (0.96–1.01)	0.99 (0.95–1.04)	NA	NA	NA		1.01 (0.88–1.17)
**March 2011 new SIV recommendations**									
Immediate change after March 2011 (2011/12)	NA	NA	NA	NA	1.02 (0.91–1.14)	**1.40 (1.29–1.52)**	1.07 (0.96–1.19)		NA
Temporal trend^d^ (2012/13–2015/16)	NA	NA	NA	NA	1.01 (0.94–1.08)	1.02 (0.97–1.07)	1.01 (0.94–1.07)		NA
**April 2013 new SIV recommendations**									
Immediate change after April 2013 (2013/14)	NA	NA	NA	NA	NA	NA	NA		**1.42 (1.14–1.78)**
Temporal trend^d^ afterwards (2014/15–2015/16)	NA	NA	NA	NA	NA	NA	NA		1.07 (0.90–1.27)

CI: confidence interval; GEE: generalised estimating equation; NA: not applicable; RR: relative risk; SIV: seasonal influenza vaccination.

^a ^Results from segmented GEE log-binomial regressions.

^b ^Persons with these conditions were recommended to be vaccinated in the 2011 SIV recommendations.

^c ^Persons with these conditions were recommended to be vaccinated in the 2013 SIV recommendations.

^d ^Temporal trend = average relative change of SIV standardised uptake rate per season over the defined period.

^e ^For liver diseases: temporal trend from 2011/12–2012/13.

RRs < 1 represent a decrease in SIV standardised uptake rates; RRs > 1 represent an increase in SIV standardised uptake rates. RRs in bold are significantly different from one.

#### Groups not concerned by new recommendations in 2011 or 2013

Immediately after the pandemic started, SIVU rates increased significantly among people ≥ 65 years old (+ 5%) and among those aged < 65 years with diabetes (+ 6%), respiratory diseases (+ 9%) and cardiac insufficiency/valvular heart diseases (+ 9%). During the season after the pandemic, rates decreased steeply in all groups (from 12 to 25%, depending on the group) and remained relatively stable until the last season (slight erosion of -2% per season in people aged ≥ 65 years, Table 2).

#### Groups concerned by the new recommendations in 2011 or 2013

The start of the pandemic was associated with a significant increase in SIVU rates in the group with coronary heart diseases ( + 9%) but not in the other three groups (strokes, neuromuscular diseases, or liver diseases), for which new recommendations would be issued in 2011 and 2013. The end of the 2009 pandemic was followed by a significant decrease among people aged < 65 years with coronary heart diseases (-13%), strokes (-16%), and neuromuscular diseases (-20%).

### Publication of new recommendations 

After the new SIV recommendations were published in March 2011 and April 2013, SIVU rates increased significantly only among the people aged < 65 years with coronary heart diseases (+ 40%) and those with chronic liver diseases (+ 42%) (Table 2) that is the two groups for which vouchers were simultaneously offered with the new recommendations. For people with neuromuscular diseases or a past strokes, who had been receiving the NHIF voucher several years prior to the publication of the recommendations, the SIVU rate did not change after their publication. 

## Discussion

### Main results

In the 2015/16 season, the crude SIVU rate was 48% in the ≥ 65 year-old group, while in the groups younger than 65 years, it ranged from 16% to 29%, according to condition. After a significant increase in SIVU during the 2009/10 pandemic season in some target groups, a steep decrease occurred in most groups during the next season followed by relative stabilisation in groups unaffected by new recommendations (a slight erosion in people aged ≥ 65 years). A significant increase in SIVU in the season immediately after the publication of new recommendations occurred only in patients with coronary heart diseases or liver diseases.

### Study limitations and strengths

Vaccinations that took place during occupational medicine visits, vaccination centres or nursing homes, which buy the vaccines for their residents are not reported to the health-related administrative databases of the French NHIF; this resulted in underestimating the coverage rate. However, these limitations are unlikely to affect the trend analysis substantially as the vast majority of vaccinations in France, for all vaccines and all ages, are administered through the private sector, and there is no reason to believe that the small proportion of vaccination delivered outside the private sector has varied over the study period. A strength of our study is that vaccine delivery data are more reliable than self-reported vaccination behaviour in surveys [20]. Indeed, although delivery of the vaccine at the pharmacy does not always convert into actual vaccination, the proportion of at-risk individuals making a successful effort to obtain the vaccine and not using it is likely to be low [9]. Another strength is that the EGB is a representative sample large enough to estimate vaccination coverage in most of the groups at risk of influenza, over a long period of time. Finally, we built on algorithms published by the NHIF [14] to identify the persons in the target groups for influenza vaccination as exhaustively as possible, by using information about LTI status, and both the delivery of drugs and diagnoses during episodes of hospitalisation associated with these diseases. Our study was thus able to include between 22% and 76% additional persons, compared with the numbers identified by tracking routine vouchers (Supplement 1). The NHIF principally uses LTIs to identify people who will be sent vouchers, although doctors do not always apply for LTI status for their patients. For example, fewer than 70% of patients treated pharmacologically for diabetes have LTI status for it, even though the pharmacologic treatments considered are specific for this disease [9]. We cannot rule out the possibility that the algorithms we used could have produced false positives, but the raw estimates of SIV coverage obtained here are probably closer to reality than those obtained with the routine algorithms.

### Seasonal influenza vaccination uptake rates far below targets

Our results show SIVU rates very far from the target in all at-risk groups, especially in those younger than 65 years. We share this disappointing observation with many other countries, but our estimates for metropolitan France are particularly low, despite the availability of free vaccine vouchers [2]. As explained above, however, a portion of the people at risk younger than 65 years do not receive the free vouchers from the NHIF and must then follow a complex pathway to be vaccinated. Moreover, they are not always informed by their physicians that SIV is recommended because of their disease [21]. Finally, multiple cognitive factors (e.g. perceptions of vaccine effectiveness and risks, beliefs related to SIV, trivialisation of the disease) can explain reluctance about SIV [21], but they are beyond the scope of this article.

### Impact of the 2009 influenza A(H1N1) pandemic

This study confirmed a modest SIV peak during the 2009 A(H1N1) pandemic in five of the eight at-risk groups considered in this study (from + 5% to + 9%). During the pandemic, officials encouraged the population to be vaccinated against both seasonal and pandemic influenza.

The clear inversion of the trend during the 2010/11 season in most of the at-risk groups and the relative stability of the SIVU rate over the next few seasons for most of them may be explained in part by the controversies that took place in France over the mass vaccination campaign in 2009 against pandemic influenza. They concerned especially its large size, its organisation, the initial non-involvement of general practitioners, the allegations of conflicts of interest of WHO expert advisors [22], the apparently excessive speed with which the vaccine was produced, and fears that it had not been adequately tested. The perception that the effects of the 2009 pandemic had been exaggerated constitute a supplementary explanation [22,23]. These controversies resulted in discrediting the French health authorities and the information they disseminated about SIV. But the lasting disaffection for this vaccination can also be linked to media coverage of the scientific debate about its effectiveness, which has grown in recent years [24-26].

### Impact of the new recommendations

The absolute value of the SIVU rate increased, albeit modestly, during the vaccination season after the publication of the new recommendations among patients with liver and coronary heart diseases. It probably expresses the effect of the voucher (which these two groups received for the first time) rather than any effect associated with the actual publication of the recommendation. This conclusion is based on the finding that the SIVU rate did not change after this publication among people with neuromuscular diseases or a past stroke, who had been receiving this NHIF voucher for several years. These results suggest that the publication of recommendations for SIV alone is not sufficient to modify vaccination behaviour, probably because general practitioners and specialists require time to absorb and apply these recommendations into their practices. New recommendations must be accompanied by complementary measures. The automatic delivery of a free voucher for the SIV is one such measure and appears to have a favourable effect. Our results, however, show a ceiling to this effect and suggest that it is not enough.

## Conclusion

Beyond the essential need for research to develop better influenza vaccines, the strategy in France to convince the public of the effectiveness and safety of the currently available SIV should be thoroughly reviewed. Interventional research in this field remains a priority. Indeed, according to Dubé et al., strong evidence to recommend any specific intervention for vaccine hesitancy/refusal is lacking in the literature, and few interventions have directly been targeted at vaccine-hesitant individuals [27]. Convincing data indicate that automated reminders to doctors and patients can significantly improve vaccination coverage by minimising the organisational work of monitoring vaccination calendars [28]. Aside from these broader issues, further improvement of SIV accessibility is essential in France. The free provision of the vaccine is a necessary but not a sufficient condition, and it is complex to implement systematically when targeted at at-risk groups. The voucher system misses numerous people targeted by the recommendations (between 22% and 76% additional persons according to the chronic diseases we studied), as mentioned in this study. Efforts have been made to simplify the vaccination pathway in France, but it remains complicated for those people at risk who do not receive the voucher. To further simplify this pathway, influenza vaccination at private community pharmacies will be generalised from 2019 [29], after an experiment of this procedure in two then four French regions respectively in 2017 and 2018, and reports of its effectiveness in other countries [30].
